# Systems Biology Studies of Adult *Paragonimus* Lung Flukes Facilitate the Identification of Immunodominant Parasite Antigens

**DOI:** 10.1371/journal.pntd.0003242

**Published:** 2014-10-16

**Authors:** Samantha N. McNulty, Peter U. Fischer, R. Reid Townsend, Kurt C. Curtis, Gary J. Weil, Makedonka Mitreva

**Affiliations:** 1 The Genome Institute at Washington University, St. Louis, Missouri, United States of America; 2 Division of Infectious Diseases, Department of Medicine, Washington University School of Medicine, St. Louis, Missouri, United States of America; 3 Department of Cell Biology and Physiology, Washington University School of Medicine, St. Louis, Missouri, United States of America; 4 Division of Endocrinology, Metabolism and Lipid Research, Department of Medicine, Washington University School of Medicine, St. Louis, Missouri, United States of America; University of Melbourne, Australia

## Abstract

**Background:**

Paragonimiasis is a food-borne trematode infection acquired by eating raw or undercooked crustaceans. It is a major public health problem in the far East, but it also occurs in South Asia, Africa, and in the Americas. *Paragonimus* worms cause chronic lung disease with cough, fever and hemoptysis that can be confused with tuberculosis or other non-parasitic diseases. Treatment is straightforward, but diagnosis is often delayed due to a lack of reliable parasitological or serodiagnostic tests. Hence, the purpose of this study was to use a systems biology approach to identify key parasite proteins that may be useful for development of improved diagnostic tests.

**Methodology/Principal Findings:**

The transcriptome of adult *Paragonimus kellicotti* was sequenced with Illumina technology. Raw reads were pre-processed and assembled into 78,674 unique transcripts derived from 54,622 genetic loci, and 77,123 unique protein translations were predicted. A total of 2,555 predicted proteins (from 1,863 genetic loci) were verified by mass spectrometric analysis of total worm homogenate, including 63 proteins lacking homology to previously characterized sequences. Parasite proteins encoded by 321 transcripts (227 genetic loci) were reactive with antibodies from infected patients, as demonstrated by immunoaffinity purification and high-resolution liquid chromatography-mass spectrometry. Serodiagnostic candidates were prioritized based on several criteria, especially low conservation with proteins in other trematodes. Cysteine proteases, MFP6 proteins and myoglobins were abundant among the immunoreactive proteins, and these warrant further study as diagnostic candidates.

**Conclusions:**

The transcriptome, proteome and immunolome of adult *P. kellicotti* represent a major advance in the study of *Paragonimus* species. These data provide a powerful foundation for translational research to develop improved diagnostic tests. Similar integrated approaches may be useful for identifying novel targets for drugs and vaccines in the future.

## Author Summary

Paragonimiasis is a food-borne trematode infection that people acquire when they eat raw or undercooked crustaceans. Disease symptoms (including cough, fever, blood in sputum, etc.) can be similar to those observed in patients with tuberculosis or bacterial pneumonia, frequently resulting in misdiagnosis. Although the infection is relatively easy to treat, diagnosis is complicated. Available diagnostic assays rely on total parasite homogenate to facilitate the detection of *Paragonimus*-specific antibodies in patients. Though these blot-based assays have shown high sensitivity and specificity, they are inconvenient because total parasite homogenate is not readily available. This study used next generation genomic and proteomic methods to identify transcripts and proteins expressed in adult *Paragonimus* flukes. We then used sera from patients infected with *P. kellicotti* to isolate immunoreactive proteins, and these were analyzed by mass spectrometry. The annotated transcriptome and the associated proteome of the antibody immune response represent a significant advance in research on *Paragonimus*. This information will be a valuable resource for further research on *Paragonimus* and paragonimiasis. Thus this project illustrates the potential power of employing systems biology for translational research in parasitology.

## Introduction

Paragonimiasis is an important food-borne trematode infection (and a “neglected tropical disease”) that is caused by lung flukes in the genus *Paragonimus* [1]–[3]. More than 50 *Paragonimus* species have been described, and nine species are known to infect humans. Human infections are most frequent in Asia (*P. westermani, P. skrjabini, P. heterotremus, P. siamensis, P. miyazakiki*), but they also occur in sub-Saharan Africa (*P. uterobilateralis, P. africanus*), and in the Americas (*P. kellicotti*, *P. mexicanus*) [1]. Approximately 21 million people are infected with *Paragonimus* worms [2], and some 293 million live in endemic areas where they are at risk of contracting the infection [3].

*Paragonimus* metacercariae enter the human host upon ingestion of raw or undercooked crustaceans. Metacercariae excyst, migrate out of the intestine, cross the diaphragm into the pleural space, and eventually invade the lungs where they mature and live for years in pulmonary cysts [1]. This results in a range of clinical symptoms, including cough, fever, weight loss, pleural effusion, chest pain, and bloody sputum [4]. These symptoms can be very similar to those seen in patients with tuberculosis, bacterial pneumonia, fungal infections, or lung cancer, so misdiagnosis is common [5]–[7]. For example, one study in the Philippines found *P. westermani* eggs rather than acid-fast bacilli in sputum samples from 26 of 160 (16%) patients with suspected tuberculosis [5]. Even in the US, the median time between onset of symptoms and diagnosis of recent *P. kellicotti* infections was approximately 12 weeks (range 3–38 weeks), and all of the patients were subjected to multiple, unnecessary medical interventions tailored to un-related diseases [8]. Once a proper diagnosis is made, parasites are easily cleared by a short course of the anthelmintic drug praziquantel, but infections can be fatal if left untreated [9].

*Paragonimus* infections are most often diagnosed by identification of parasite eggs in the stool or sputum (reviewed in [1]). Unfortunately, migrating parasites are capable of causing disease weeks or months before eggs production commences. Egg detection is also insensitive due to temporal inconsistencies and requires knowledge and expertise that are not readily available in many clinical settings. Serological tests for *P. westermani* and *P. kellicotti* using native parasite antigens have been described, but these tests are impractical for widespread use because they require continued access to adult parasites [8], [10], [11]. Thus far, efforts to develop and implement practical, standardized molecular diagnostic tools have been hindered by a lack of information on the basic biology and genomics of *Paragonimus* species.

**Figure 1:**
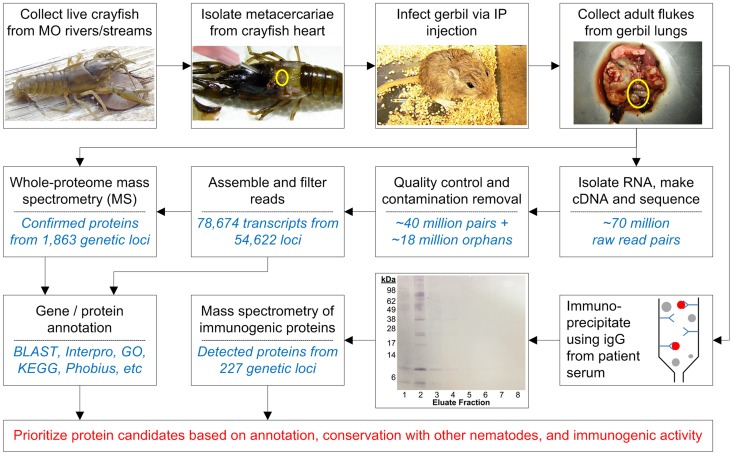
Characterization of the adult transcriptome, adult proteome, and immunogenic proteins of *Paragonimus kellicotti*.

According to the study outline presented in Figure 1, we sequenced and annotated the transcriptome of adult *P. kellicotti* to better understand this parasite at a molecular level and to facilitate proteomic analyses of both the total worm homogenate and of immunogenic proteins purified using IgG from *P. kellicotti* patient sera. The resulting sequence data led to the identification of proteins that are promising candidates for the development of novel (and much needed) serodiagnostic tests for paragonimiasis. In addition, the annotated transcriptome of adult *P. kellicotti* provides a valuable resource for molecular biological and translational research on paragonimiasis and related food-borne trematode infections.

## Materials and Methods

### Parasite material

Wild crayfish (genus *Orconectes*)>3 cm in length were collected from small rivers in southern Missouri, USA. *P. kellicotti* metacercariae, identified by morphological examination, were isolated from the hearts of infected crayfish and introduced to Mongolian gerbils (*Meriones unguiculatus*) by intraperitoneal injection as previously described [12]. Gerbils were sacrificed 35–49 days post-infection, and egg-producing adult flukes were removed from lung cysts, rinsed in 1× phosphate buffered saline (PBS), and stored at −80°C prior to use in experiments.

### RNA isolation and sequencing

Total RNA was isolated from two mature adult flukes using the PureLink RNA Mini Kit according to the manufacturer's microcentrifuge pestle protocol for animal tissues (Ambion, Austin, TX), and DNase treated using the TURBO DNA-free Kit (Ambion). cDNA was synthesized and sequenced as previously described [13]. Briefly, poly(A) RNA was selected from total RNA using the MicroPoly(A) Purist Kit (Ambion) and reverse transcribed using the Ovation RNA Amplification System V2 (NuGEN Technologies, Inc., San Carlos, CA). Paired-end, small fragment, Illumina libraries with insert sizes ranging from 180–380 bp were constructed and sequenced on an Illumina HiSeq2000 version 3 flow cell according to the manufacturer's recommended protocol (Illumina Inc., San Diego, CA). Raw reads were deposited in the NCBI sequence read archive under accession number SRX530756 (NCBI BioProject Accession: PRJNA179523).

### RNAseq read processing and assembly

Raw reads were converted from bam to fastq format using Picard Tools' SamToFastq script (http://picard.sourceforge.net). cDNA synthesis and Illumina sequencing adapters were trimmed using Flexbar [14] and Trimmomatic [15], respectively. Trimmomatic was also used to perform sliding window quality trimming (5 bp window, average quality ≥20) and removal of reads less than 60 consecutive high quality bases and reads containing ambiguous base calls [15]. Reads with an average DUST score less than seven were removed using the filter_by_complexity script from the seq_crumbs package (http://bioinf.comav.upv.es/seq_crumbs/). Remaining reads were mapped against ribosomal RNA [16], [17] and bacterial sequence databases [18] with Bowtie2 (version 2.1.0, default parameters, [19]) and against the human genome (hs37) and GenBank rodent database (gbrod, downloaded April 24, 2013) with Tophat2 (version 2.0.8, default parameters, [20]); all matching reads and their mates were excluded from further analysis. The remaining high quality *P. kellicotti* originated reads were assembled using the Trinity *de novo* RNAseq assembler [21] with default parameters. Modules within the Trinity software package were used to estimate transcript abundance and remove transcripts representing <1% of the per-component expression level and <1 transcript per million [21], [22]. The RNAseq reads used for the assembly were re-mapped to the high-confidence transcripts with Bowtie2 (version 2.1.0, default parameters, [19]) and transcript breadth of coverage (defined as the percent of covered bases over the length of the reference transcript) was assessed using RefCov (http://gmt.genome.wustl.edu/genome-shipit/gmt-refcov/current/). Transcripts with <99% breadth of coverage with RNAseq reads were removed, resulting in the final transcript set. Assembly statistics at each phase of filtering are given in Table S1. It is expected that the *de novo* assembly would over-estimate the number of transcripts and loci, so in-house PERL scripts were used to estimate fragmentation based on WU-BLAST alignments to protein coding sequences from closely related species as previously described [23]. Assembly fragmentation was calculated as the percentage of reference genes associated with multiple, non-overlapping BLAST hits.

### Transcriptome annotation

All assembled transcript isoforms were compared to known protein sequences by BLASTX [24] against the GenBank Non-Redundant protein database (NR, downloaded April 15, 2014). Results were parsed to consider only top matches to non-overlapping regions of the query with e-value less than 1e-05. Putative protein translations from the transcripts were predicted using Prot4EST [25]. Transmembrane domains and secretion peptides were predicted using Phobius [26], [27]. Proteins were assigned to KEGG orthologus groups, biochemical pathways and pathway modules using KEGGscan [28] with KEGG release 68. Associations with known InterPro domains and Gene Ontology (GO) classifications were inferred from predicted protein sequences using InterProScan [29]–[31]. Functional enrichment of GO terms was calculated using FUNC with an adjusted p-value cutoff of 0.01 [32]. For FUNC analysis, the target list included the longest isoform of a given locus that contained the feature of interest against the background of the longest isoforms of all loci including the target list. All transcripts, predicted proteins, and associated annotations are available at Trematode.net (trematode.net/Paragonimus_kellicotti.html).

### Preparation and fractionation of adult parasite antigen

Adult parasite antigen was prepared as previously described [12]. Briefly, eight adult parasites were homogenized on ice in RIPA buffer (10 mM Tris-HCl, pH 7.4, 150 mM NaCl, 1% NP-40, 0.2% sodium deoxycholate, 1 mM EDTA and 10 mM NaF) using a 1 mL mini homogenizer (GPE Scientific Limited, Leighton Buzzard, UK). The homogenate was centrifuged at 19,000×g for 15 minutes and the supernatant was collected. Protein concentration was measured using the Pierce BCA assay kit (Thermo Scientific, Rockford, IL), and 500 µg was loaded onto GELFrEE 8100 fractionation system with an 8% cartridge (Expedeon, San Diego, CA) [33], [34]. Eight molecular weight fractions were collected and the proteins were precipitated using a modified acetone-based method as previously described [35]. The pellets were solubilized in Tris buffer (100 mM Tris-HCl pH 8.5) containing 8M urea and the protein content was determined using the Advanced Protein Assay (Cytoskeleton, Inc., Denver, CO) [(Fraction 1 (F1, lowest molecular weight), 35 µg; F2, 176 µg; F3, 126 µg; F4, 83 µg; F5, 71 µg; F6, 67 µg; F7, 76 µg; F8, 40 µg)]. The quality of molecular weight fractionation was analyzed by SDS-PAGE; proteins were labeled with Sypro Ruby, and results were scanned using a Typhoon 9400 instrument.

### Immunoprecipitation and purification of *P. kellicotti* proteins

De-identified serum samples from *P. kellicotti* infected patients were obtained from Barnes Jewish Hospital in St. Louis, Missouri, the Centers for Disease Control and Prevention in Atlanta, GA, and Heartland Medical Center in St. Joseph, MO. Patients included in this study had reported ingestion of raw crayfish, exhibited symptoms consistent with paragonimiasis, tested positive for *Paragonimus* exposure using existing serological or parasitological diagnostic assays, and had no recent history of international travel. In all cases, sera were collected prior to treatment.

Patient sera were tested for reactivity against adult *P. kellicotti* and *P. westermani* antigen by Western blot as previously described [10]. Serum samples from five strongly-reactive patients were pooled (total volume 3 mL), and total IgG was precipitated using saturated ammonium sulfate (Thermo Fisher Scientific, Pittsburg, PA), re-suspended in 1× phosphate buffered saline (PBS), and desalted by dialysis against 4L 1× PBS for 2 hours at room temperature, against 4L 1× PBS 2 hours at 4°C, and against 4L 1× PBS overnight at 4°C.

Two mL Pierce NHS-active agarose slurry (Thermo Fisher Scientific) was added to a 2.0 mL spin column (Thermo Fisher Scientific), and rinsed with 2.0 mL water followed by 2.0 mL 1× PBS. Two mL of IgG precipitated from the paragonimiasis serum pool was added to the column and mixed for 2 hours at room temperature to couple IgG to the agarose. The column was washed once with 1× PBS, blocked with 1.0M ethanolamine pH 7.4 for 20 minutes at room temperature, and washed again with 1× PBS.

Approximately 720 mg of adult *P. kellicotti* total antigen was added to the column and incubated overnight at 4°C. Column was washed with 1× PBS, and immune complexes were eluted with Pierce IgG elution buffer (Thermo Scientific) in eight 1 mL fractions. Fractions were neutralized with 50 µL 1.0M Tris, pH 9.0, and 10 µL aliquots of each fraction were analyzed by Western blot as previously described using the pooled patient sera as the primary antibody [10]. The fraction with the highest concentration was precipitated using the 2D clean-up kit (GE Healthcare, Buckinghamshire, UK) and the pellet was solubilized in 20 µL 100 mM Tris-HCl, pH 8.5 with 8M urea to prepare peptides for mass spectrometry.

### Digestion of proteins for mass spectrometry

The proteins that were eluted and denatured from the antibody coupled beads or from the GELFrEE protein fractions were reduced with 1 mM TCEP (Pierce) for 30 min, and alkylated with 20 mM Iodoacetamide (Sigma) at room temperature in the dark for 30 min. The reaction was quenched with 10 mM DTT (Sigma) for 15 min. Endoprotease Lys-C (Sigma) (5 µg) was added and the samples were digested in a barocycler (Pressure Biosciences) [36] for 30 min at 37°C, followed by dilution to 2M urea with the Tris buffer, addition of trypsin (Sigma) and barocycler digestion for 30 min at 37°C. The digest was acidified to 5% formic acid and peptides were desalted in parallel on Glygen Nutips containing C4 and graphite carbon solid phase on a Beckman Biomek (Biomek NXP), as previously described [37]. The eluted peptides were dried in a SpeedVac and dissolved in water/acetonitrile/formic acid (99%/1%/1%) and transferred to autosampler vials (SUNSRI Cat No. 200-046) for storage at −80°C or LC-MS analysis.

Peptides for LC-MS from the GELFrEE fractionation were prepared as described above with the following modification. The endoprotease digests were acidified to 1% TFA, filtered through a 30K MWCO filter (Sartorius VIVACON 500). Peptides were desalted on a SepPak cartridge (50 mg/1cc) (Waters), dried in a SpeedVac and transferred into the autosampler vials for LC-MS analysis.

### Liquid chromatography, tandem mass spectrometry (LC-MS/MS) analysis and mapping

A NanoLC 2D Plus System with a cHiPLC-Nanoflex and AS2 autosampler (Eksigent, Dublin,CA) was configured with two columns in parallel. One cHiPLC column (ChromXP C_18_ (200 µm×15 cm; particle size 3 µm, 120 Å) was used to inject calibrant solution (β-galactosidase peptides (625 pmol/vial, part# 4333606) and another cHiPLC column was used for sample analysis. The calibrant solution (500 fmol) was injected in solvent A (water/formic acid/AcN, 98%/1%/1%). The samples were loaded in a volume of 10 µL at a flow rate of 0.8 µL/min followed by gradient elution of peptides at a flow rate of 800 nL/min. The calibrant solution was eluted with the following gradient conditions with solvent B (water/formic acid/AcN, 1%/1%/98%):0, 2%; 3 min, 2%; 73 min, 50%; 83 min, 80%; 86 min, 80%; 87 min 2%; 102 min, 2%. The digests from the immune-affinity purified samples were analyzed under the following gradient conditions (time, percent solvent B): 0, 2%; 3 min, 2%; 205 min, 35%; 215 min, 80%; 240 min, 2%. The digests from the GELFrEE fractionation were analyzed under the following gradient conditions (time, percent solvent B): 0, 2%; 5 min, 2%; 650 min, 35%; 695 min, 80%; 700 min, 2%; 720 min, 2%.

Data acquisition was performed with a TripleTOF 5600+ mass spectrometer (AB SCIEX, Concord, ON) fitted with a Picoview Nanospray source (PV400)(New Objectives, Woburn, MA) and a 10 µm Silica PicoTip emitter (New Objectives, Woburn, MA). Data were acquired using an ion spray voltage of 2.9 kV, curtain gas of 20 PSI, nebulizer gas of 25 psi, and an interface heater temperature of 175°C. The MS was operated with a resolution of greater than or equal to 25,000_fwhm_ for TOFMS scans. For data dependent acquisition, survey scans were acquired in 250 mS from which 100 product ion scans were selected for MS2 acquisition for a dwell time of 20 mS. Precursor charge state selection was set at +2 to +5. The survey scan threshold was set to 100 counts per second. The total cycle time was fixed at 2.25 seconds. Four time bins were summed for each scan at a pulser frequency value of 15.4 kHz through monitoring of the 40 GHz multichannel TDC detector with four-anode/channel detection. A rolling collision energy was applied to all precursor ions for collision-induced dissociation using the equation 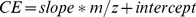, where the slope for all charges above 2+ is 0.0625 and the intercept is −3,−5 and −6 for 2+,3+, and 4+, respectively.

The raw LC-MS data (*.wiff) were converted to *.mzML format utilizing the AB SCIEX MS Data Converter v 1.3 (AB SCIEX, Foster City, CA) within PEAKS STUDIO 7.0 (Bioinformatics Solutions Inc., Waterloo, Canada). The resulting files were used for database searching by the PEAKS software using protein translations from the *P. kellicotti* transcriptome. The Ensembl Human protein database (Homo_sapiens.GRCh37.72) was used to identify human background proteins in the sample matrix. The searches were conducted with trypsin cleavage specificity, allowing 3 missed cleavages, oxidation of Met and carbamidomethylation of Cys as variable and constant modifications, respectively. A parent ion tolerance of 25 ppm and a fragment ion tolerance of 100 milli-mass units were used. The MS2-based peptide identifications were validated within PEAKS software using a modified target decoy approach, decoy fusion, to estimate the FDR [38]. A 1% FDR for peptide spectral matches was used as the quality filter to identify peptides and associated proteins. MS data are available from Trematode.net (trematode.net/Paragonimus_kellicotti.html) and PeptideAtlas (identifier PASS00555).

### Ethics statement

All animal work was performed in compliance with relevant US and international guidelines. Animal studies protocols were approved by the Washington University School of Medicine Animal Studies Committee (Animal Welfare Assurance # A-3381-01). The Animal Studies Committee complies with the United States Public Health Service Policy for Humane Care and Use of Laboratory Animals and other standards as required by the NIH Office of Laboratory Animal Welfare. The use of anonymized human sera was approved by the Washington University in St. Louis Institutional Review Board (DHHS Federal Assurance #FWA00002284) under approval number 201102546.

## Results/Discussion

### Characterizing the adult transcriptome of *P. kellicotti*

Prior to this study, a total of 911 GenBank sequences were available from the genus *Paragonimus*, only seven of which were from *P. kellicotti*. Therefore, it was necessary to sequence, assemble and analyze the transcriptome of *P. kellicotti* to enable further study (Table 1). Approximately 70 million paired-end reads were generated from an adult *P. kellicotti* cDNA library on the Illumina HiSeq platform. Following removal of low quality and contaminant reads, 40 million read pairs and 18 million unpaired orphan reads were assembled into 78,674 high-confidence transcript isoforms with an average length of 560 bp. These were further clustered into 54,622 distinct genetic loci, 21.5% of which are associated with more than one transcript isoform (mean 3.0 transcript isoforms per alternatively spliced locus). We assume that the *P. kellicotti* genome contains a similar number of protein coding genes as other recently sequenced trematode genomes, which currently ranges from 10,852 in *Schistosoma mansoni* to 16,258 in *Clonorchis sinenesis* [39]–[43]. The discordance between the number of detected genetic loci and the expected number of genes is likely due to assembly fragmentation resulting in overestimation of the number of genes, a common problem seen in *de novo* transcriptome assemblies of short read data [44]–[46]. We calculated the fragmentation rate of our assembly at 25.8% using *S. mansoni* genes as a reference and at 31.4% using *C. sinensis* genes as a reference. The fragmentation rate is an estimate and it depends on the level of sequence conservation between the species of interest and species with available genome data; however, it is likely that at least 25.8–31.4% of all *P. kellicotti* genes represented in our assembly are split into two or more non-overlapping genetic loci.

**Table 1:** *Paragonimus kellicotti* transcriptome assembly statistics.

**Raw Data**	
Raw sequence reads	69,874,039 pairs
Cleaned, decontaminated reads	39,564,722 pairs & 17,866,916 orphans
**Assembly**	
Transcript isoforms	78,674
Average transcript length	560 bp
Genetic loci	54,622
AS loci	11,771
Average isoforms per AS loci	3.04
**Predicted Proteins**	
Unique proteins	77,123
Average protein size	113 aa
Transcripts with associated protein	78,663
Loci with associated protein	54,616
**Annotation**	
InterPro domains	4,407
GO terms	1,234
KEGG orthologous groups	6,854
KEGG pathways	336
KEGG pathway modules	284

Assembled transcripts were compared to known proteins originating from other species. A total of 32,201 transcript isoforms from 20,102 loci shared a sequence similarity with an e-value cut-off of better than 1e-05 (Table S2). A majority of the matches were to sequences from *C. sinensis* followed by *Schistosoma* species. This is not surprising, as these were the only trematodes with sequenced genomes at the time this study was conducted. *P. kellicotti* sequences shared an average 61.3% sequence identity with corresponding *C. sinensis* sequences at the protein level. There were just 165 *P. westermani* sequences included in GenBank-NR at the time of this study, so only 125 transcripts from 67 genetic loci had a top BLASTX hit to a *P. westermani* protein. The sequence identity shared between *P. kellicotti* and *P. westermani* high-scoring segment pairs was 79.8% at the protein level. *P. kellicotti* and *P. westermani* are not considered to be close relatives within the genus *Paragonimus* [47]; however, the identified high level of sequence conservation may help facilitate the design of pan-*Paragonimus* serological assays.

**Figure 2:**
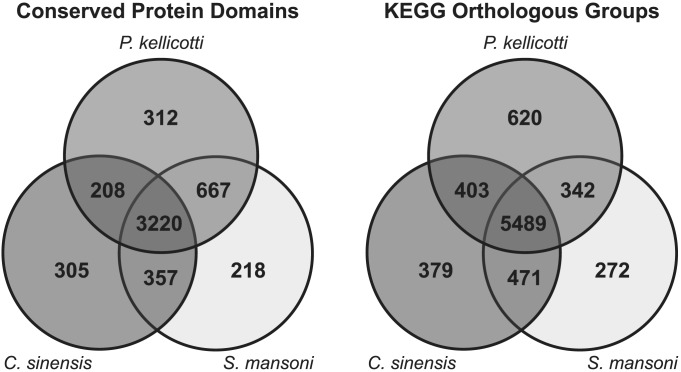
Distribution of protein sequence similarity matches among three trematode species. Venn diagram showing the distribution of (**A**) InterPro protein domains and (**B**) KEGG orthologous groups shared or unique to *P. kellicotti*, *C. sinensis* and *S. mansoni*.

A total of 77,123 unique protein sequences were predicted from 54,616 of the detected genetic loci. Detailed annotations are available in Table S2. Predicted proteins from 11,116 genetic loci were associated with a total of 4,407 unique InterPro protein domains and 1,234 unique GO terms. The number of genetic loci associated with each molecular function term was tallied, and the most abundantly represented terms were related to protein, ATP and nucleic acid binding. Similarly, the biological processes with the highest representation were protein phosphorylation, metabolic process, and oxidation-reduction process. In a comparison between three trematode species, a total of 312 conserved domains were unique to *P. kellicotti*, while 305 and 218 were unique to *C. sinensis* and *S. mansoni*, respectively (Figure 2A). A majority of the domains present in each species were shared between all three species.

Predicted proteins from 18,028 transcripts/11,599 genetic loci were associated with 6,854 unique KEGG orthologous groups. These were further binned into 336 unique biochemical pathways and 284 pathway modules. The KEGG orthologous groups represented in the adult of transcriptome of *P. kellicotti* were compared to those represented in the draft genomes of *C. sinensis* and *S. mansoni* (Figure 2B). Altogether, 620 *P. kellicotti* KEGG orthologous groups (KOs) were absent from the other trematodes; these were binned into 255 pathways and 97 modules, most of which were very sparsely populated with the *P. kellicotti*-specific KOs. A careful analysis failed to identify any complete or nearly complete pathways present in *P. kellicotti* but absent in the other trematodes. The coverage of specific KEGG pathways can be visualized and compared to other trematodes using the TremaPath tool available at Trematode.net (http://trematode.net/TN_frontpage.cgi?navbar_selection=comparative_genomics&subnav_selection=tremapath).

Secreted proteins have an important role in the life cycle of tissue-migrating parasite species like *P. kellicotti*, facilitating interactions with the host. These proteins are of practical interest as diagnostic, vaccine, or drug targets. Proteins related to 1,610 genetic loci were annotated as potentially secreted based on the presence of a classical signal peptide for secretion and absence of a predicted transmembrane domain (Table S2). Seven GO terms were found to be enriched among predicted secreted proteins, with the most highly enriched term being related to cysteine protease activity (Table 2). Proteases tend to be prevalent among trematode excretory-secretory products [48]–[51], and various reports have described their role in migration through host tissues, nutrient uptake, and immune evasion [52]–[55].

**Table 2:** Functions enriched among proteins with predicted secretion peptides.

Root	GO Term	Description	Corrected p-value
Molecular Function	GO:0004197	cysteine-type endopeptidase activity	1.99E-08
Biological Process	GO:0006508	proteolysis	0.0001
Molecular Function	GO:0004867	serine-type endopeptidase inhibitor activity	0.0009
Biological Process	GO:0045454	cell redox homeostasis	0.001
Cellular Component	GO:0009331	glycerol-3-phosphate dehydrogenase complex	0.001
Molecular Function	GO:0051537	2 iron, 2 sulfur cluster binding	0.008
Cellular Component	GO:0005615	extracellular space	0.009

### Characterizing the adult worm proteome of *P. kellicotti*

Total parasite antigen was subjected to analysis by mass spectrometry to survey the worm proteome and subsequently to validate a subset of our assembled transcripts. A total of 244,048 spectra were matched to 25,405 database protein predictions that corresponded to 2,555 transcripts from 1,863 genetic loci (Table S2). The verified proteins encompass 1,626 InterPro protein domains, 586 GO terms, 1,925 KEGG orthologous groups from 307 pathways and 198 pathway modules. Furthermore, 63 transcripts from 48 genetic loci with no annotation (i.e., no significant BLAST hit in NR or KEGG, conserved protein domain, GO term, etc.) were confirmed by the proteomic data. These sequences, thus far unique to *P. kellicotti*, might have otherwise been dismissed as low confidence transcripts due to the draft nature of the transcriptome assembly. However, proteomic evidence verified that these species-specific nucleotide sequences are translated and that they may have important biological functions in *P. kellicotti*.

In order to obtain an estimate of abundance, identified proteins were ranked according to associated spectral counts. Given the draft nature of the transcriptome and the known issue of fragmentation, attempts were not made to correct for protein size, so follow up experiments would be required to assess abundance in a more robust and quantitative manner. The 25 proteins with highest spectral counts (Table 3) included actins, myoglobins, chaperone proteins, and yolk ferritins, and these proteins may be abundant in the parasites. Oxygen binding proteins such as myoglobin are vital to parasite survival, as an exceptionally high affinity for their substrate allows the parasite to scavenge oxygen from host blood and tissues [56]. The high abundance of myoglobin proteins in our analysis may serve as an indication of the importance of aerobic respiration in *P. kellicotti*.

**Table 3:** The top 25 *Paragonimus kellicotti* proteins in adult worms based on spectral counts.

Transcript	Top BLAST Hit	Unique Peptides	Spectral Count
Pk39535_txpt1	*Paragonimus westermani* myoglobin 2 (gi:59895955, 4e-91)	592	7206
Pk29718_txpt2	*Fasciola hepatica* Fatty acid-binding protein type 3 (gi:47116941, 1e-55)	466	6465
Pk37407_txpt1	*Clonorchis sinensis* glutamate dehydrogenase (NAD(P)+) (gi:358253764, 0.0)	263	3658
Pk42024_txpt1	*Paragonimus westermani* 28 kDa glutathione-S transferase (gi:2264324, 3e-106)	311	3496
Pk02080_txpt2	*Schistosoma mansoni* actin (gi:256084605, 0.0)	212	2692
Pk45213_txpt1	*Crassostrea gigas* Actin-2 (gi:405973339, 0.0)	209	2690
Pk34178_txpt1	*Paragonimus westermani* myoglobin 1 (gi:59895953, 5e-91)	238	2589
Pk48313_txpt1	*Paragonimus westermani* yolk ferritin (gi:13625997, 1e-77)	114	2301
Pk33942_txpt3	*Clonorchis sinensis* glyceraldehyde 3-phosphate dehydrogenase (gi:349917947, 7e-165)	143	2238
Pk37138_txpt1	*Caenorhabditis elegans* Protein ACT-4, isoform a (gi:17568985, 0.0)	150	1962
Pk47122_txpt1	*Clonorchis sinensis* fructose-bisphosphate aldolase class I (gi:358332246, 0.0)	144	1849
Pk47113_txpt2	*Clonorchis sinensis* mitochondrial malate dehydrogenase (gi:47531133, 0.0)	129	1765
Pk29799_txpt3	*Schistosoma mansoni* cysteine synthase (gi:256071387, 3e-162)	122	1631
Pk37388_txpt3	*Clonorchis sinensis*tyrosine 3-monooxygenase/tryptophan 5-monooxygenase activation protein (gi:358339010, 4e-119)	105	1574
Pk02531_txpt1	*Clonorchis sinensis* molecular chaperone HtpG (gi:358339046, 0.0)	116	1567
Pk47362_txpt2	Clonorchis sinensis chaperonin GroEL (gi:358255039, 3e-161)	133	1533
Pk02081_txpt1	*Clonorchis sinensis* actin beta/gamma 1 (gi:358339578, 1e-140)	127	1465
Pk08185_txpt1	*Fasciola gigantica* heat shock protein 70 (gi:153861697, 0.0)	90	1454
Pk42528_txpt2	*Paragonimus westermani* yolk ferritin (gi:13625997, 3e-67)	104	1408
Pk24292_txpt1	*Schistosoma japonicum* thioredoxin peroxidase-2 (gi:60279643, 2e-104)	84	1383
Pk48312_txpt2	*Paragonimus westermani* yolk ferritin (gi:13625997, 2e-83)	115	1314
Pk42696_txpt1	*Clonorchis sinensis* propionyl-CoA carboxylase alpha chain (gi:358255536, 0.0)	81	1215
Pk52615_txpt1	*Fasciola hepatica* protein disulphide isomerase (gi:3392892, 9e-104)	67	1157
Pk27756_txpt1	*Ancylostoma ceylanicum* hypothetical protein (gi:597857576, 4e-140)	78	1154
Pk34236_txpt1	*Echinostoma caproni* enolase (gi:112950027, 0.0)	97	1145

Protein abundance was estimated by un-corrected spectral counts. Only the top-scoring transcript from each genetic locus was considered in ranking the top 25 most abundant proteins as long as the isoforms had similar top BLAST hits and annotations. The GenBank accession number of the top BLAST match and the e-value of the match are given in parentheses.

### Identification of potential serodiagnostic antigens using antibodies from patient sera

**Figure 3:**
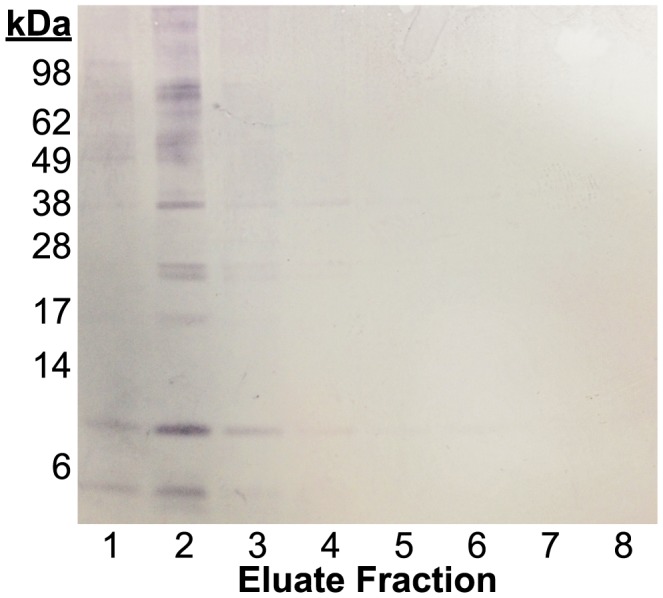
Western blot of *Paragonimus kellicotti* antigen immunoprecipitated with total IgG from *P. kellicotti* patients. Total IgG was purified and used to precipitate immunogenic proteins from total *P. kellicotti* homogenate. *P. kellicotti* proteins were eluted from the purification column in eight fractions, which were tested by Western blot using an aliquot of the same IgG used in the immunoprecipitation. Fraction 2 had the greatest protein concentration and was used in our mass spectrometry analysis.

Serodiagnostic assays based on worm homogenate have been shown to sensitively and specifically detect an immune response to *P. westermani* and *P. kellicotti* [8], [10], [11]. In these assays, total parasite protein antigens are analyzed by SDS PAGE gel electrophoresis, transferred to a membrane, and exposed to patient serum. Doublet bands appearing at 21/23 kDa and a more diffuse band at 34 kDa are indicative of exposure to *Paragonimus* species (Figure 3 and [10]). However, the identity of these proteins was not known.

An unusual cluster of cases of paragonimiasis (caused by *P. kellicotti*) occurred in recent years in the state of Missouri [8], [57], [58]. Since helminth infections are uncommon in Missouri, sera from these patients contain antibodies to *Paragonimus* antigens, but they are unlikely to contain antibodies to antigens of other helminths. These sera represented an excellent resource for our study. *P. kellicotti* proteins recognized by total IgG from some of these patients were enriched by immunoprecipitation using affinity beads. Eluate fractions were assessed by Western blot (Figure 3), and the strongest fraction was analyzed by mass spectrometry. A total of 2,406 spectra were matched to 1,443 proteins predicted from the transcriptome assembly that corresponded to 321 transcripts from 227 genetic loci (Table S2). Some 212 of these 227 loci were also detected in our analysis of the total worm proteome. Thus, the whole parasite proteome provided useful supplementary information to the immunoprecipitated proteins. The 25 most abundant proteins bound by patient IgG (as approximated by spectral counts) are listed in Table 4. Most of the translations predicted from the transcriptome represent a fraction of the full length of the deduced protein. Therefore, it is challenging to determine with certainty which of these might represent the antigen present in the 21/23 kDa or 34 kDa bands. Nonetheless, several of the proteins on this list are of interest as potential serodiagnostic antigens.

**Table 4:** The top 25 immunoreactive *Paragonimus kellicotti* proteins in adult worms based on spectral counts.

Transcript	Top BLAST hit	Unique Peptides	Spectral Count	Predicted Secretion Peptide
Pk00394_txpt2	*Paragonimus westermani* cysteine protease 6 (gi:67773374, 0.0)	50	112	yes
Pk45107_txpt2	*Clonorchis sinensis* cystatin-2 (gi:150404782, 1e-43)	33	88	no
Pk48549_txpt1	*Paragonimus westermani* cysteine protease 8 (gi:67773378, 0.0)	35	73	
Pk34206_txpt1	*Schistosoma mansoni* ATP synthase alpha subunit mitochondrial (gi:256070850, 0.0)	37	64	no
Pk45997_txpt1	*Schistosoma mansoni* ATP synthase beta subunit (gi:256077755, 0.0)	27	52	no
Pk34178_txpt1	*Paragonimus westermani* myoglobin 1 (gi:59895953, 5e-91)	27	50	no
Pk45213_txpt1	*Crassostrea gigas* Actin-2 (gi:405973339, 0.0)	27	43	no
Pk07379_txpt2	*Clonorchis sinensis* legumain, partial (gi:358331503, 1e-177)	26	42	no
Pk02080_txpt2	*Schistosoma mansoni* actin (gi:256084605, 0.0)	27	41	no
Pk24571_txpt1	*Clonorchis sinensis* putative leucyl aminopeptidase (gi:118767252, 0.0)	23	39	no
Pk50870_txpt1	*Clonorchis sinensis* elongation factor-1 (gi:46410394, 0.0)	21	37	no
Pk45998_txpt1	*Schistosoma japonicum* ATP synthase, H+ transporting, mitochondrial F1 complex, beta polypeptide (gi:226487054, 8e-102)	16	34	no
Pk53261_txpt2	no hit	17	32	no
Pk39524_txpt1	*Fasciola hepatica* MF6p protein, partial (gi:379991184, 5e-16)	10	32	no
Pk24292_txpt1	*Schistosoma japonicum* thioredoxin peroxidase-2 (gi:60279643, 2e-104)	15	31	no
Pk29718_txpt2	*Fasciola gigiantica* Fatty acid-binding protein type 3 (gi:47116941, 1e-55)	15	30	no
Pk48295_txpt2	*Clonorchis sinensis* peptidase inhibitor 16 (gi:358338291, 2e-35)	15	28	no
Pk49950_txpt1	Paragonimus westermani unknown protein (gi:13625983, 8e-74)	17	27	no
Pk52615_txpt1	*Fasciola hepatica* protein disulphide isomerase (gi:3392892, 9e-104)	13	25	no
Pk49951_txpt2	*Paragonimus westermani* unknown protein (gi:13625983, 6e-111)	7	25	no
Pk42039_txpt2	*Paragonimus westermani* pre-procathepsin L (gi:2731635, 1e-142)	15	23	yes
Pk52616_txpt1	*Clonorchis sinensis* protein disulfide-isomerase A1, partial (gi:358256495, 3e-93)	13	23	yes
Pk39535_txpt1	*Paragonimus westermani* myoglobin 2 (gi:59895955, 4e-91)	11	23	no
Pk01058_txpt1	*Clonorchis sinensis* molecular chaperone DnaK (gi:358336042, 0.0)	16	22	no
Pk02081_txpt1	*Clonorchis sinensis* actin beta/gamma 1 (gi:358339578, 1e-140)	12	22	no

Protein abundance was estimated by un-corrected spectral counts. Only the top-scoring transcript from each genetic locus was considered in ranking the top 25 most abundant proteins as long as the isoforms had similar top BLAST hits and annotations. The GenBank accession number of the top BLAST match and the e-value of the match are given in parentheses. The presence or absence of a predicted secretion peptide is noted in the table; however, there are many routes of release from a live worm (both active and passive) that do not involve a classical secretion signal.

Five of the highly abundant immunoreactive proteins (Table 4), Pk00394_txpt2, Pk45107_txpt2, Pk48549_txpt1, Pk24571_txpt1, and Pk42039_txpt2 are putative cysteine proteases. Translations from three of these transcripts (Pk00394_txpt2, Pk48549_txpt1, and Pk42039_txpt2) are predicted to have molecular weights in the range of 35–36 kDa, close in size to the diffuse ∼34 kDa antigen detected by serodiagnostic Western blots with total native parasite antigen (Figure 3). The predictions of 35–36 kDa are only estimates and may not represent the full length of the protein. However, the predicted molecular weights of top BLASTX hits of these proteins are in the same size range (36–37 kDa), and this indicates that the *P. kellicotti* sequences we have are complete or nearly so. Recombinant cysteine proteases have shown promise as serodiagnostic antigens for trematode infections [59]–[63], and a previous study reported that partially purified cysteine proteases from *P. westermani* excretory-secretory products were superior for antibody diagnosis compared to whole worm antigen extracts [64]. Two of the most abundant proteins identified in the mass spectrometry analysis of our *P. kellicotti* immunoprecipitate, Pk00394_txpt2 and Pk48549_txpt1, share 86% sequence identity at the amino acid level. These proteins are similar to cysteine proteases from other *P. westermani* and, to a lesser extent, helminths of other genera. By selecting a specific region from these cysteine proteases, it may be possible to develop an assay that discriminates between *Paragonimus* species and other helminths. A recombinant cysteine protease from *P. westermani*, rPwCP2, has already shown promise as diagnostic antigen [62], but this sequence (gi:42516556) has no homolog in our *P. kellicotti* transcriptome. Thus, the cysteine proteases identified in our study may be more useful as a pan-*Paragonimus* diagnostic reagent than those previously described.

**Figure 4:**
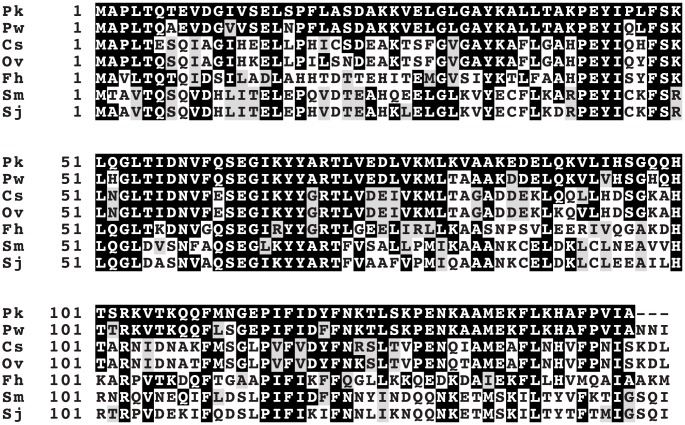
Alignment of myoglobin proteins from *Paragonimus* species and other trematodes. Amino acid alignments show 90% sequence identity between myoglobin 1 sequences from *P. kellicotti* and *P. westermani*. Far less homology is shared between myoglobins in *Paragnomimus* and top BLASTP hits from other trematode species. Abbreviations: Pk1, Pk34178_txpt1; Pk2, Pk48549_txpt1; Pw, *P. westermani* gi:59895953; Cs, *C. sinensis* gi:349998765; Ov, *Opisthorchis viverrini* gi: 663047528; Fh, *F. hepatica* gi:159461074; Sm, *S. mansoni* gi:256084837; Sj, *S. japonicum* gi:226487206.

Other proteins on our top-25 list (Table 4), such as the MF6p proteins and myoglobins, have not been considered as serodiagnostic antigens, but they are abundant excretory-secretory products of trematodes and merit further exploration. For example, Pk39524_txpt1 is annotated as a putative MF6p protein. Its top BLAST hit was recently characterized as a heme-binding protein and is a major antigen secreted by *F. hepatica* [65]. The *P. kellicotti* orthologue only shares 57% sequence identity with the *F. hepatica* protein, so cross-reactivity with antibodies in patients with fascioliasis should not be a major problem. Orthologs from other *Paragonimus* species have not yet been reported, so it is not possible to assess the potential utility of this protein as a pan-genus diagnostic reagent at this time. However, Pk34178_txpt1, a putative myoglobin 1, shares 90% sequence identity with an ortholog in *P. westermani*, but significantly less similarity with orthologs from other trematode species (Figure 4), strongly indicating that this candidate is worth further attention to examine its diagnostic utility.

### Conclusions

We undertook a systems biology approach to comprehensively study the adult transcriptome and proteome of *P. kellicotti* to improve understanding of the protein composition of the adult parasite and potential interactions between the parasite and its mammalian host. The transcriptome of adult *P. kellicotti* represents a major advance in the study of *Paragonimus* species. Transcriptomes provide powerful foundations for translational research in parasitology to develop improved diagnostic tests, treatments, and vaccines. In this study, transcriptome data was used together with immunoaffinity chromatography and mass spectrometry to efficiently identify candidate diagnostic antigens. Similar integrated approaches may be useful for identifying novel targets for drugs and vaccines. Finally, the data generated in this study (transcriptome, proteome, and immunolome) represent a valuable resource for the research community, and it will be especially helpful for annotating genomes of *Paragonimus* spp. as they become available.

## Supporting Information

Table S1 **Transcriptome assembly statistics at various stages of filtering.** Information is provided on the content and completeness of the *P. kellicotti* transcriptome assembly after each filtering step.
(DOCX)

Table S2 **Annotation of the** ***P. kellicotti*** **transcriptome assembly.** Information on the annotation of assembled transcripts is provided here. This includes the top NR BLASTX hit, InterPro protein domains, gene ontology terms, KEGG orthologous groups, biochemical pathways, pathway modules, transmembrane domains and secretion signals. The numbers of MS peptides and spectral counts associated with each transcript are also provided.
(DOCX)
