# Supplementary material for: Systems Biology Studies of Adult Paragonimus Lung Flukes Facilitate the Identification of Immunodominant Parasite Antigens
Source: PLoS Negl Trop Dis. 2014 Oct 16;8(10):e3242. doi: 10.1371/journal.pntd.0003242 (PMC4199545; doi:10.1371/journal.pntd.0003242)
Supplement: Table S1 — Transcriptome assembly statistics at various stages of filtering. Information is provided on the content and completeness of the P. kellicotti transcriptome assembly after each filtering step. (DOCX) [file pntd.0003242.s001.docx]

**Table S1. Transcriptome assembly statistics at various stages of filtering.**

|  | Unfiltered | RSEM filtered* | Refcov 99%** | Refcov 100% |
| --- | --- | --- | --- | --- |
| Genetic loci | 153,461 | 59,050 | 54,622 | 48,832 |
| Transcripts | 251,721 | 91,029 | 78,674 | 65,135 |
| Average transcript length | 460 bp | 563 bp | 560 bp | 538 bp |
| Read pairs mapped | 68.3% | 66.3% | 64.33% | 59.24% |
| InterPro domains | 4,888 | 4,538 | 4,401 | 4,170 |
| GO terms | 1,357 | 1,261 | 1,234 | 1,195 |
| BLASTx aubjects | 23,556 | 16,549 | 15,393 | 13,848 |
| Alternative splicing | 24.8% AS, ave 3.58, max 85 | 24.4%, ave 3.2, max 20 | 21.5% AS, 3.04 ave, max 17 | 18.1% AS, 2.8 ave, 14 max |

*Scripts included in the Trinity software package were used to remove transcripts representing <1% of the per-component expression level and < 1 transcript per million.

**This version of the assembly was chosen for further analyses and described in the manuscript.
